# Pseudogenization of NK3 homeobox 2 (*Nkx3.2*) in monotremes provides insight into unique gastric anatomy and physiology

**DOI:** 10.1098/rsob.240071

**Published:** 2024-07-03

**Authors:** Jackson Dann, Zhipeng Qu, Linda Shearwin-Whyatt, Rachel van der Ploeg, Frank Grützner

**Affiliations:** ^1^ School of Biological Sciences, University of Adelaide, Adelaide, SA 5005, Australia

**Keywords:** pseudogenization, homeoboxes, *Nkx3.2*, monotremes, evolution, development

## Abstract

The enzymatic breakdown and regulation of food passage through the vertebrate antral stomach and pyloric sphincter (antropyloric region) is a trait conserved over 450 million years. Development of the structures involved is underpinned by a highly conserved signalling pathway involving the hedgehog, bone morphogenetic protein and Wingless/Int-1 (Wnt) protein families. Monotremes are one of the few vertebrate lineages where acid-based digestion has been lost, and this is consistent with the lack of genes for hydrochloric acid secretion and gastric enzymes in the genomes of the platypus (*Ornithorhynchus anatinus*) and short-beaked echidna (*Tachyglossus aculeatus*) . Furthermore, these species feature unique gastric phenotypes, both with truncated and aglandular antral stomachs and the platypus with no pylorus. Here, we explore the genetic underpinning of monotreme gastric phenotypes, investigating genes important in antropyloric development using the newest monotreme genomes (mOrnAna1.pri.v4 and mTacAcu1) together with RNA-seq data. We found that the pathway constituents are generally conserved, but surprisingly, NK3 homeobox 2 (*Nkx3.2*) was pseudogenized in both platypus and echidna. We speculate that the unique sequence evolution of *Grem1* and *Bmp4* sequences in the echidna lineage may correlate with their pyloric-like restriction and that the convergent loss of gastric acid and stomach size genotypes and phenotypes in teleost and monotreme lineages may be a result of eco-evolutionary dynamics. These findings reflect the effects of gene loss on phenotypic evolution and further elucidate the genetic control of monotreme stomach anatomy and physiology.

## 1. Introduction

Monotremes (order Monotremata) evolved unique changes in their gastric anatomy and physiology since their divergence from therian mammals 187 million years ago. The platypus (*Ornithorhynchus anatinus*) and the short-beaked echidna (*Tachyglossus aculeatus*) are two of the few vertebrate species to have lost acidic gastric juices and glandular antral epithelium [1]. Changes in diet and ecological specialization since their divergence 55 million years ago have led to the morphological evolution of the gastrointestinal tract between these two monotremes. The platypus stomach is small, amorphic, glandless and it lacks a pyloric sphincter, making it notably hard to distinguish from the oesophagus and intestines. The echidna stomach, while also glandless and non-acidic, is bulbous, containing a pyloric-like restriction through to the duodenum to regulate the flow of food and gastric juices [2–4].

The antral stomach and pyloric sphincter (antropyloric region) are shared features throughout vertebrate evolution, which can be dated back to early diverging jawed-vertebrate lineages around 450 million years ago. Monotremes are one of the few lineages that have lost these structures, making them an interesting species to investigate the molecular signatures associated with this change in digestive anatomy. The glandular antral stomach is responsible for the secretion of hydrochloric acid to maintain the acidic luminal environment, while the pyloric sphincter regulates the passage of food into the duodenum and prevents acidic juices from damaging duodenal epithelium. The development of this structure is controlled by a highly conserved developmental pathway found in the zebrafish, chicken, frog and mouse [5,6].

**Figure 1.**
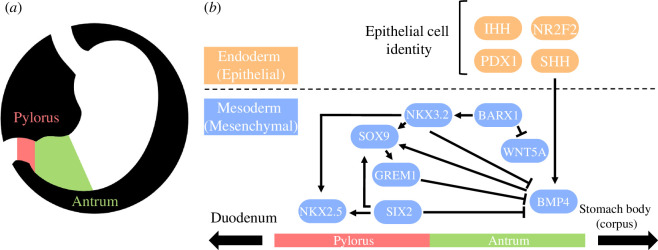
Diagrammatic representation of the stomach anatomy (*a*) and the conserved vertebrate antropyloric developmental pathway (*b*). Constituents of the pathway are divided according to their roles in epithelial and mesenchymal development (orange and blue in *b*) and their positioning along the horizontal axis depicts whether they are implicated in pyloric and/or antral development. Adapted from Self *et al*. [10] and Udager *et al*. [5].

A key gene of this pathway, the NK3 homeobox 2 (*Nkx3.2*), has epithelial and mesenchymal roles in antropyloric development as well as other roles in axial and peripheral skeletal development [7,8]. During stomach development, *Nkx3.2* expression in undifferentiated gastric mesenchyme demarcates the future pylorus from the antral stomach and duodenum by repressing *Bmp4* expression and potentiating *Nkx2.5*, *Sox9*, *Six2* and *Grem1* expression. The *Barx1-Nkx3.2* signalling cascade at this timepoint also represses Wnt signalling (especially *Wnt5a*) to promote antral stomach identity [7–9]. Development of gastric epithelial cell identities such as gastrin-releasing G cells in the antral stomach and pylorus is regulated by the interaction of sonic hedgehog protein (encoded by *Shh*) and *Nkx3*.2. Other proteins such as Indian hedgehog protein (*Ihh*), nuclear receptor subfamily 2 group F member 2 (*Nr2f2*) and pancreatic-and-duodenal homeobox 1 (*Pdx1*) potentiate epithelial cell type development using many additional pathways (figure 1) [11–13].

Here, we present the expression of antropyloric developmental genes in monotremes and conservation in a range of vertebrates and identify *Nkx3.2* to have been pseudogenized in the monotreme ancestor. We then speculate further on what changes may be associated with morphological differences between the platypus and echidna and outline overlapping eco-evolutionary dynamics between monotremes and other vertebrate lineages which have lost gastric acid digestion.

## 2. Material and methods

### 2.1. Sequence retrieval, alignments and phylogenies

Protein products of *Nkx3.2*, *Barx1, Shh*, *Ihh, Pdx1, Nr2f2, Nkx2.5, Six2, Bmp4, Sox9, Grem1* and *Wnt5a* were acquired through NCBI GenBank (https://www.ncbi.nlm.nih.gov/genbank/). Sequences were obtained from eutherian mammals (human, *Homo sapiens*; house mouse, *Mus musculus*; brown rat, *Rattus norvegicus*; horse, *Equus caballus*; cow, *Bos taurus*; pig, *Sus scrofa*), marsupials (common wombat, *Vombatus ursinus*; gracile agile opossum, *Gracilinanus agilis*), reptiles (chicken, *Gallus gallus*; fence lizard, *Sceloporus undulatus*), bony fishes (zebrafish, *Danio rerio*) and cartilaginous fishes (salmon, *Salmo salar*). Sequences for monotremes (platypus, *Ornithorhynchus anatinus*; short-beaked echidna, *Tachyglossus aculeatus*) were acquired from the newly released genome sequences: mOrnAna1.pri.v4 and mTacAcu1.pri respectively [14].

Multiple sequence alignments of derived protein sequences were performed using the Clustal Omega plugin in Geneious Prime 11.0.14+1 [15,16]. Protein phylogenies were constructed using the online IQ-TREE web server with ModelFinder for the substitution model and UFBOOT for 1000 bootstrap replicates. Amino acid substitution models were chosen based on Bayesian information criterion log maximum-likelihood estimates [17–19]. Substitutions, insertions and deletions occurring in conserved domains that occurred exclusively in one or both of the monotreme species were then noted and compared with the associated literature or the conserved domain database [20].

### 2.2. Expression analysis

RNA-Seq fastq files for platypus and echidna tissues were mapped to the platypus genome mOrnAna1.pri.v4 and mTacAcu1.pri using HISAT2 [14,21]. Gene expression for *Nkx3.2* and other pathway genes were analysed using FeatureCounts 2.0.0 and RSEM 1.3.3 with default settings, respectively. For *Nkx3.2* expression analysis, exon coordinates were manually annotated in GTF format and random intergenic regions were chosen as a negative control for transcriptional noise [22–24].

### 2.3. gDNA/RNA preparation and cDNA synthesis

Snap-frozen monotreme liver, stomach and spleen samples stored at −80°C were lysed using liquid nitrogen in a mortar and pestle. Monotreme liver gDNA was prepared using the phenol-chloroform method [25]. RNA samples from monotreme stomach, liver and spleen were prepared using the Qiagen RNeasy plus micro kit (Qiagen, Hilden, Germany) as per instructions. Total RNA samples from monotreme tissues were reverse transcribed using the iScript cDNA synthesis kit (Bio-Rad, Hercules, CA, USA). RNA was stored in nuclease-free water at −80°C.

### 2.4. Polymerase chain reaction

β-Actin (*Actb*) and NK3 homeobox 2 (*Nkx3.2*) primers were designed using the NCBI primer-blast (https://www.ncbi.nlm.nih.gov/tools/primer-blast/) and synthesized by integrated DNA technologies (Coralville, IA, USA) (table 1).

**Table 1.** Species, genes and primer sequences used for gDNA PCRs and RT-PCRs.

species	gene	primer sequence	annealing temperature (°C)
platypus (*O. anatinus*) and echidna (*T. aculeatus*)	β-actin (*Actb*)	F: 5′ GCC CAT CTA CGA AGG TTA CGC 3′ R: 5′ AAG GTC GTT TCG TGG ATA CCA C 3′	57
echidna (*T. aculeatus*)	*Nkx3.2*	F: 5′ AAG GCT CGG AAG AAG CGT 3′ R: 5′ GGA GGC AGT AAT AGG GGT AGT A 3′	60
platypus (*O. anatinus*)	*Nkx3.2*	F: 5′ CCA CAG TGG ATC GCA GCC 3′ R: 5′ TCT TGT AGC GCC GGT TCT GGA A 3′	65

Genomic DNA and cDNA polymerase chain reactions (PCRs) were carried out using OneTaq polymerase (New England Biolabs, Ipswich, MA, USA) as per instructions. Each 25 μl reaction contained 5 μl GC reaction buffer, 0.5 μl 10 mM dNTPs, 1 μl forward/reverse primer 10 μM mix, 0.25 μl polymerase and 18.25 μl nuclease-free water. Cycling occurred in the C1000 touch thermal cycler (Bio-Rad). Conditions involved an initial denaturation at 94° for 3 min, then 35 cycles of 30 s denaturation at 94°, 30 s annealing at appropriate temperatures (table 1) and extension at 68° for 1 min kb^−1^. All PCR products were confirmed by Sanger sequencing (Australian Genome Research Facility, Melbourne, Victoria, Australia).

## 3. Results

### 3.1. Sequence changes and expression of antropyloric developmental genes

To determine the conservation of epithelial and mesenchymal antropyloric developmental genes, monotreme amino acid sequences were aligned to those of a range of vertebrate species and changes to sequence structure or conserved domains likely to affect structure, function or processing were noted. Gene expression in monotremes was investigated using RNA-seq datasets, and PCR products were compared either with non-transcribed region controls or benchmark transcript per million (TPM) standards (electronic supplementary material, table S1).

All genes were expressed in at least one tissue beyond a significant threshold (>0.5 TPM) except *Pdx1* where expression was ambiguous in monotreme tissues (electronic supplementary material, table S1). Monotreme *Bmp4* sequences contained several short (9–15 amino acids) insertions N-terminal to and within the signalling domain, respectively, and 7–14 amino acid insertions N-terminal to the second splice site [26]. The echidna *Grem1*-derived protein sequence contained an N-terminal insertion of 16 amino acids, and both sequences shared several missense mutations in the *Bmp4* inhibitory domain not conserved elsewhere (electronic supplementary material, table S1) [27].

### 3.2. Vestigial *Nkx3.2* sequences remain in the conserved syntenic region in monotremes

As a key gene implicated in epithelial and mesenchymal development of the antropyloric region, we sought to examine its conservation and expression in monotreme species [5]. Using conserved vertebrate domains from Lettice *et al*. [28], some sequence remnants were identified in monotreme genomes. To ensure the sequences identified were associated with *Nkx3.2*, synteny analysis was performed. In the six vertebrate species analysed (human, opossum, platypus, echidna, chicken and lizard), we found strongly conserved syntenic blocks flanking either side of the *Nkx3.2* locus. Reconstructed monotreme *Nkx3.2* sequences occurred in the conserved syntenic region between *Rab28* and *Bod1l1* (electronic supplementary material, figure S1). Given a lack of annotations, intron–exon boundaries were then estimated using conserved vertebrate motifs from Lettice *et al*. [28], and average conserved exon lengths from other mammalian taxa (electronic supplementary material, figure S1).

**Figure 2.**
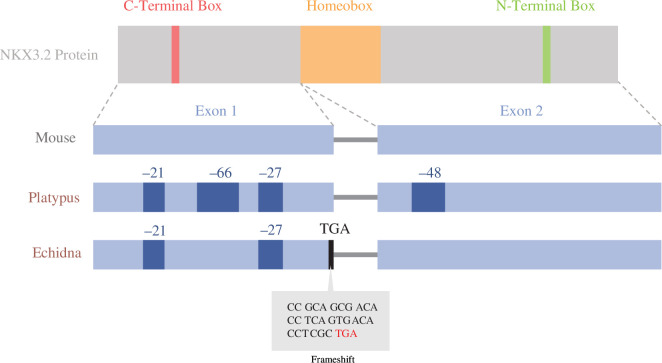
Visualization of *Nkx3.2* protein and exon architecture in the mouse, platypus and echidna overlayed with inactivating mutations and deletions found in monotreme taxa. Dark blue patches represent large deletions, black lines represent premature stop codons and the grey box represents a nucleotide alignment of the inactivating mutation in the echidna: a stop codon emerging from a frameshift mutation. Adapted from Osipova *et al*. [29].

Monotreme *Nkx3.2* DNA and amino acid sequences were then aligned to determine significant sequence changes in conserved domains (figure 2). The identities of reconstructed sequences ranged between 37% and 55% when compared with those of vertebrates, which was lower than other vertebrate sequences with each other, ranging from 50% to 95% sequence identity. The lower-than-expected sequence identities in monotremes may be attributed to an accumulation of various insertions/deletions and mutations in between the C-terminal box, homeobox and N-terminal box. These conserved domains in NKX superfamily proteins were mostly conserved between monotreme species. Reconstruction of the open reading frame using conserved vertebrate intron–exon boundaries revealed an inactivating stop codon due to a frameshift mutation in the first exon of the echidna sequence (figure 2).

### 3.3. Monotreme *Nkx3.2* shows no significant expression in adult and juvenile monotreme tissues

Major sequence changes in *Nkx3.2* suggest that this gene has mutated extensively, so we investigated its transcriptional activity. The *Nkx3.2* exon coordinates did not align with any liver RNA-seq data in NCBI, so expression was analysed using mammalian RNA-seq datasets [30]. Few reads mapped to the predicted exons of *Nkx3.2* from a variety of platypus and echidna tissues (table 2), and most reads mapped could be corroborated with intergenic controls indicating transient, non-significant expression (table 3).

**Table 2.** Number of reads mapped to exon coordinates for *Nkx3.2* in various tissues from the two monotreme species and the resulting RPKM values.

	tissue	mapped read total for gene	mapped read total for tissue	RPKM
echidna	adult ovary	0	54 548 375	0
	adult testes	0	61 299 876	0
	adult frontal cortex	0	58 422 726	0
	puggle frontal cortex	0	47 214 854	0
	puggle ovary	2	47 923 676	0.007
	puggle testes	0	54 413 890	0
platypus	brain	0	37 686 965	0
	fibroblasts	0	39 841 608	0
	kidney	0	18 380 826	0
	liver	0	38 897 053	0
	ovary	1	25 060 266	0.012
	testes	0	66 530 964	0

**Table 3.** Number of reads mapped to randomly selected non-transcribed regions in various tissues from the two monotreme species and the resulting RPKM values.

	tissue	mapped read total for gene	mapped read total	RPKM
echidna	adult ovary	0	54 548 375	0
	adult testis	1	61 299 876	0.003
	adult frontal cortex	0	58 422 726	0
	puggle frontal cortex	1	47 214 854	0.004
	puggle ovary	1	47 923 676	0.004
	puggle testis	0	54 413 890	0
platypus	brain	0	37 686 965	0
	fibroblasts	0	39 841 608	0
	kidney	0	18 380 826	0
	liver	1	38 897 053	0.001
	ovary	0	25 060 266	0
	testes	1	66 530 964	0.001

**Figure 3.**
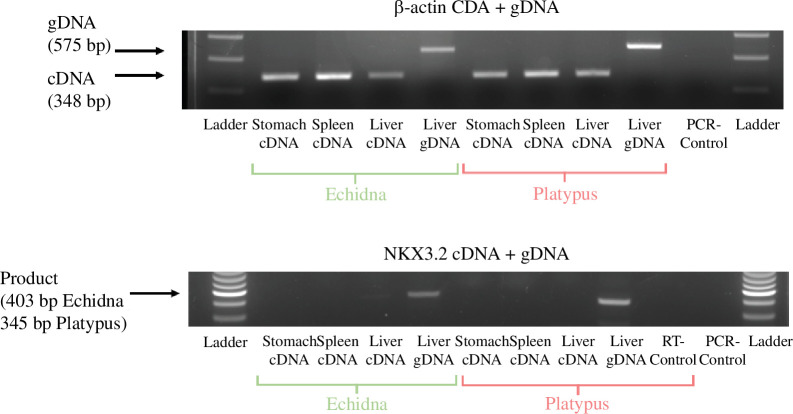
Genomic and RT-PCRs of platypus and echidna cDNA and gDNA from stomach, liver and spleen using primers for β-actin (*a*) and *NKX3.2* (*b*). Owing to high GC content and large introns, intra-exonic primers were used for *Nkx3.2* PCRs, where absence versus presence would be indicative of expression.

To verify the absence of expression, reverse-transcription PCR (RT-PCR) was carried out on platypus and echidna stomach, spleen and liver tissues. β-Actin genomic DNA and expression were used as controls in each tissue. No expression was observed (figure 3). We conclude that monotremes have lost a functional *Nkx3.2* gene.

## 4. Discussion

Since their divergence from therian mammals 187 million years ago, gastrointestinal morphology has changed significantly in echidna and platypus. There has been a loss of acidic gastric juices and antral glandular epithelium, a reduction in stomach size in the platypus, and a loss of the pylorus in the platypus despite the echidna displaying a pyloric-like restriction [1,2,5]. We investigated the highly conserved vertebrate antropyloric developmental pathway to determine the genetic basis for these contrasting phenotypes.

### 4.1. Sequence changes in antropyloric developmental genes provide clues for gastric phenotype discrepancy

Overall we found conservation of the antropyloric developmental genes *Nr2f2*, *Sox9*, *Six2*, *Wnt5a* and *Barx1*. Insertions, deletions and missense mutations in *Shh* and *Nkx2.5* were mostly conserved between monotreme species and therefore unlikely to be associated with the different gastric phenotypes. Of the epithelial genes, echidna *Pdx1* was missing the majority of the canonical PCIF-1 domain sequence. The PCIF-1 domain allows the PDX-1–PCIF-1 interface and transcriptional inhibition of PDX-1, which is necessary for pancreatic islet and duodenal development [31,32]. The echidna Indian hedgehog (*Ihh*) sequence contained a large insertion of 70 amino acids in the N-terminal signalling domain, compared to a 25 amino acid insertion in the platypus. This domain is necessary for the transduction of the hedgehog signalling pathway [33]. Of the mesenchymal genes, the echidna *Grem1* contained a unique N-terminal insertion in the signal peptide and sequence change in the bone morphogenetic protein (BMP) inhibitory domain shared with the platypus. The inhibitory domain prevents BMP receptor binding and the downstream signalling pathway, a necessary step in the regulation of many fundamental developmental processes. Echidna *Bmp4* also contained a unique missense mutation at the furin S2 cleavage site, which probably precludes activity and normal signalling of the mature peptide [26,27,34].

Surprisingly, our results show that *Nkx3.2* is not expressed in either species, and the genes have accumulated neutral mutations and a stop codon that is likely to reflect pseudogenization. This suggests a pseudogenization event in the most recent ancestor of monotremes that may correlate with previously noted deletions of gastric enzyme genes and those associated with hydrochloric acid production.

### 4.2. The *Nkx3.2* pseudogenization probably occurred in the monotreme ancestor and is probably associated with the loss of antral and pyloric segment identities

Transcriptome and RT-PCR results from this study showed an absence of *Nkx3.2* expression in any monotreme tissues. This, together with an accumulation of missense mutations not found elsewhere and a predicted premature stop codon in echidna, provides strong evidence for a shared pseudogenization that occurred in the monotreme ancestor before their divergence 55 million years ago. Pseudogenization of *Nkx3.2* in monotremes and their corresponding gastric phenotypes mirrors the phenotype of *Nkx3.2* knockout mice, which show no pyloric restriction; truncated antral sections and markers for antral glands were severely reduced and located near the duodenum or lost [35,36].

In the absence of *Nkx3.2*, the establishment of the echidna pyloric-like restriction would probably require compensatory inhibition of *Bmp4* as is seen in mouse and chicken developmental models [4]. Knockout *Six2* and *Nkx3.2* models show the additive and essential roles of both these genes in boundary formation, with *Grem1* misexpression studies showing additive inhibition of *Bmp4* expression but no effect on the pyloric phenotype [10]. Of the sequence changes listed above, the most likely candidates that may cause the gastric phenotypic difference between the platypus and echidna are *Grem1* and *Bmp4,* as they both affect the *Bmp4* expression domain and therefore establish the boundary between the pylorus and antral stomach. The insertion in the signal peptide domain of echidna *Grem1* would only affect protein translocation and secretion, which may have no effect on *Bmp4* inhibitory capacity. The missense mutations in the BMP inhibitory domain of *Grem1* may affect protein–protein interactions but are shared between monotreme species. In echidna *Bmp4*, the missense mutation in the furin S2 cleavage site may affect activity and signalling, but it is unclear whether this would also affect other aspects of development given the broad function of the protein.

### 4.3. Eco-evolutionary adaptation of the monotreme ancestor stomach

The pseudogenization and loss of gastric genes and morphology in the monotreme ancestor are also present sporadically in aquatic gnathostome lineages. Select teleost species (e.g. *Takifugu rubripes*, *Danio rerio* and *Oryzias latipes*) and Chimaeriformes species (*Callorhinchus milii*) have converged upon an agastric phenotype associated with pseudogenization or loss of the hydrochloric acid-associated and gastric enzyme genes as observed in the monotremes [5,37]. Lineages have differed in proposed selective pressures for these losses such as rudimentary gas exchange organs (*Hypostomus plecostomus*), swallowing sea water (*T. rubripes*), adaptations to diets high in calcium carbonate (*Anarhichas lupus*) or swallowing large amounts of undigestible material such as detritivores (*Mugilidae* spp.) [38,39]. Though selective pressures differ, with the exception of the terrestrial short-beaked echidna, the agastric phenotype has only been observed in aquatic/semi-aquatic vertebrate lineages.

Limited fossil, physiological and molecular evidence has led to debate about whether the platypus lineage is associated with a change from a terrestrial to a semi-aquatic ecological specialization in the monotreme ancestor or vice versa [40–42]. The semi-aquatic platypus forages at the bottom of freshwater ecosystems to store bottom-dwelling aquatic invertebrates and gravel in their cheeks, which have accessory maxillary pads for mechanical digestion of food at the water’s surface [2]. The combination of feeding behaviours is suggestive that the potential accumulation of detritus in the stomach may drive loss of an acidic gastric environment such as that found in *Mugilidae* species. If the agastric phenotype was a trait associated with aquatic/semi-aquatic organisms, then this molecular evidence would support a semi-aquatic ancestor of the short-beaked echidna and platypuswith subsequent adaptations to the echidna lineage accounting for return and specialization in terrestrial ecologies.

## 5. Conclusion

Here, we investigated the genetic underpinnings of the monotreme antropyloric region and found that all constituents of the vertebrate antropyloric pathway, with the exception of *Nkx3.2*, were largely conserved and expressed. Interestingly, the functional loss of *Nkx3.2* is consistent with losses of antropyloric segments such as the antral glandular epithelium in both species and the pylorus in the platypus. The discrepancy between the loss of pylorus in platypus and pyloric-like restriction in the echidna may be associated with independent evolution in *Grem1* and *Bmp4* sequences, but regardless, it leaves the question of whether retention of independent evolution of pyloric-like restriction occurred in the short-beaked echidna lineage. The polyphyletic presence of the agastric phenotype in aquatic and semi-aquatic teleost and monotreme lineages, with the exception of the short-beaked echidna, led us to speculate that convergent eco-evolutionary dynamics may be responsible and may offer support for a semi-aquatic ancestor of the short-beaked echidna and platypus.

## Data Availability

All sequence data are openly available through NCBI GenBank (https://www.ncbi.nlm.nih.gov/genbank/), otherwise all data necessary to evaluate results and conclusions are available within the paper, references and supplementary materials [43].
